# Resilience under pressure: a systematic review of psychological coping and endurance mechanisms among collegiate tennis athletes in higher education

**DOI:** 10.3389/fpsyg.2025.1730060

**Published:** 2026-02-09

**Authors:** Zihan Gao, Wan Ahmad Munsif Wan Pa, Mohamad Nizam Bin Nazarudin

**Affiliations:** Faculty of Education, Universiti Kebangsaan Malaysia, Bangi, Malaysia

**Keywords:** anxiety, collegiate athletes, coping mechanisms, mental fatigue, psychological resilience, stress management

## Abstract

**Objective:**

This systematic review aimed to synthesize empirical evidence on psychological resilience as a protective and adaptive mechanism for managing stress, anxiety, and mental fatigue among collegiate tennis athletes.

**Methods:**

Following PRISMA 2020 and PICOS frameworks, literature was retrieved from Scopus, Web of Science, PubMed, ERIC, SPORTDiscus, and CNKI databases. Eligibility criteria included studies published between 2010 and 2025 focusing on college-level tennis players. The review was registered with PROSPERO (CRD4202554321). Quality assessment was conducted using MMAT (2018).

**Results:**

Fifteen studies met inclusion criteria. Resilience significantly moderated the relationship between stress and fatigue, mediated anxiety through coping strategies, and predicted higher performance consistency.

**Conclusion:**

Resilience training and coping-skill interventions are essential for collegiate athletes’ psychological well-being and performance stability.

## 1 Introduction

### 1.1 Background: collegiate tennis as a complex psychosocial stress ecosystem

Collegiate sport has long been regarded as an important context for supporting students’ holistic development, offering opportunities to cultivate discipline, emotional maturity, teamwork, and leadership across competitive settings (Gayles, 2019). Within this landscape, tennis occupies a distinctive position because its competitive structure places athletes in a psychologically demanding dual role. On the court, players perform in complete isolation—making rapid tactical and technical decisions without immediate team support. Yet, paradoxically, their individual match results directly influence broader team outcomes, including institutional rankings and scholarship considerations (Kremer et al., 2020).

This convergence of personal responsibility and team consequence creates a uniquely high-pressure environment. Players must regulate their own performance while simultaneously carrying the weight of collective expectations, resulting in a level of psychological strain that exceeds what is typically observed in team-based sports. Managing these intertwined demands requires not only technical expertise but also considerable emotional resilience and adaptive coping.

In contrast to team-based sports such as soccer or basketball, collegiate tennis affords athletes virtually no opportunity for in-match communication or emotional reinforcement from teammates. Competitors must regulate their thoughts, emotions, and tactical decisions independently while navigating the inherently unstable rhythms of play—marked by rapid momentum swings, prolonged points, and minimal external feedback. Such conditions create a fertile ground for performance-related anxiety, intrusive cognitive rumination, and momentary lapses in attentional control, patterns that have been consistently documented in studies involving collegiate tennis players (Gustafsson et al., 2017).

Evidence from Asian sporting contexts reflects similar dynamics. Lim and Tan (2023), for instance, reported that tennis is perceived as one of the most psychologically demanding sports in Malaysian university athletics, surpassed only by gymnastics. When these pressures accumulate over time, athletes face increased risks of mental fatigue, reduced executive control, and a heightened likelihood of injury or burnout (Smith, 2022). These findings reinforce the importance of psychological resilience as a critical buffer that enables athletes to withstand and adapt to the chronic stressors inherent in collegiate tennis. Recent geopolitical and structural developments have further intensified the stress ecosystem surrounding collegiate tennis.

In the post-pandemic landscape, collegiate tennis programs have faced increasingly compressed competitive calendars, often requiring athletes to compete in midweek fixtures, weekend doubleheaders, and prolonged travel itineraries (Pieterse and Bester, 2024). In the United States, the implementation of Name–Image–Likeness (NIL) policies has further expanded the demands placed on student-athletes, introducing commercial obligations and heightened media exposure. These pressures carry particular weight in tennis, where individual visibility is already considerable. Comparable developments have unfolded in China’s CUBA league, where rapid commercialization has amplified expectations surrounding athlete branding and consistent high-level performance (Zhang and Wang, 2021).

Concurrently, digital transformations have reshaped the everyday realities of collegiate tennis athletes. The normalization of livestreamed practices, algorithm-driven performance analytics, and instantaneous online commentary has intensified self-surveillance and increased vulnerability to external critique (Hill et al., 2022). Hybrid academic arrangements blur the boundary between study and recovery, contributing to screen-induced fatigue and disrupted sleep patterns (Lastella et al., 2021). These challenges are compounded by persistent inequalities in institutional resources. Athletes within underfunded programs frequently lack access to sport psychology support, high-quality medical care, and advanced recovery technologies—conditions associated with greater risk of burnout and prolonged distress (Fletcher and Sarkar, 2012).

Additionally, the internationalization of collegiate tennis presents its own set of pressures. Approximately one-third of NCAA Division I tennis athletes come from abroad, and many must simultaneously manage linguistic barriers, cultural transitions, and unfamiliar academic norms (Ryba et al., 2020).

Taken together, these factors illustrate that collegiate tennis functions as a complex psychosocial ecosystem shaped by digital hyper-exposure, academic techno-strain, cultural adjustment demands, and structural inequalities. Understanding how athletes navigate these intersecting pressures—and how psychological resilience enables adaptation within this environment—is critical for supporting sustainable performance, mental well-being, and long-term educational success.

### 1.2 Defining psychological resilience and coping: conceptual foundations

Early conceptualizations of psychological resilience framed it as a relatively stable dispositional trait—commonly referred to as hardiness—characterized by perseverance, commitment, and a general tolerance for stress (Kobasa, 1979). Over the past two decades, however, resilience has been increasingly understood as a dynamic, contextually embedded process shaped by the interplay of cognitive, emotional, biological, and social systems (Windle, 2011; Masten, 2018). Luthar and Cicchetti’s (2000) seminal definition—“positive adaptation within the context of significant adversity”—captures this process-oriented orientation. Within the sport domain, resilience reflects an athlete’s capacity to draw upon internal strengths and external resources to regulate stress, sustain confidence, and recover effectively from performance disruptions (Fletcher and Sarkar, 2012).

Coping strategies represent a central mechanism through which resilience becomes observable in competitive settings. According to Lazarus and Folkman’s (1984) Transactional Model, athletes navigate stress through two appraisal processes: an initial evaluation of the situational demands (primary appraisal) followed by an assessment of available coping resources (secondary appraisal). Resilient athletes are more likely to appraise stressors as challenges rather than threats, a framing that promotes the use of adaptive coping behaviors. Self-Determination Theory (Deci and Ryan, 2000) offers an important complementary perspective by suggesting that resilience is strengthened when athletes experience autonomy, competence, and relatedness—conditions shown to reduce anxiety and enhance self-regulatory effectiveness (Gucciardi et al., 2018).

Coping itself comprises a range of cognitive and behavioral strategies. Problem-focused coping involves modifying or eliminating the stressor (e.g., adjusting tactics or training plans), whereas emotion-focused coping aims to regulate internal emotional states (e.g., cognitive reframing, controlled breathing). Empirical research consistently demonstrates that strategies such as imagery, structured self-talk, pre-performance routines, and attentional refocusing are associated with lower competitive anxiety and more stable performance under pressure (Nicholls and Polman, 2007). Conversely, avoidant coping—characterized by withdrawal, denial, or disengagement—has been strongly linked to burnout risk and diminished resilience over time (Rice et al., 2016).

The related construct of psychological endurance extends traditional resilience frameworks by emphasizing athletes’ capacity to sustain functional stability under prolonged or cumulative stress exposures (Jones et al., 2019). This perspective highlights resilience not merely as a momentary response but as an ongoing regulatory process that supports adaptive functioning across extended competition periods.

Neuroscientific research increasingly supports this multidimensional conceptualization. Functional MRI studies indicate that resilient athletes exhibit heightened connectivity across neural regions involved in executive control and affect regulation, including the anterior cingulate cortex, insula, and prefrontal cortex (Hamlin et al., 2023). These neural signatures provide a biological explanation for the documented efficacy of mindfulness-based interventions, cognitive-behavioral strategies, and structured mental-skills training—all of which enhance top-down modulation of stress responses and foster more adaptive psychological functioning in competitive environments.

### 1.3 Integrating coping models and neurocognitive mechanisms

The Transactional Model of Stress and Coping continues to offer a central lens for understanding how athletes appraise and manage stress in competitive environments. Nicholls and Polman (2007) demonstrated its particular relevance to sport, showing that higher-performing athletes tend to adopt more flexible and adaptive coping strategies, including reinterpreting stressors as challenges and employing deliberate emotion-regulation techniques.

Folkman (1987) emphasis on positive emotions further enriched this framework by introducing the concept of challenge appraisal, a view closely aligned with Fredrickson’s (2001) Broaden-and-Build Theory. According to this model, positive emotions broaden cognitive and attentional capacities, enabling athletes to generate more creative solutions and accumulate psychological resources that support long-term resilience.

Neurocognitive research lends empirical support to these propositions. Enhanced activation within the anterior cingulate cortex (ACC) and dorsolateral prefrontal cortex (DLPFC) has been associated with greater attentional stability and cognitive flexibility—capacities that are indispensable in tennis, where shifting match dynamics and constantly evolving tactical demands require rapid, precise decision-making.

Interventions such as Mindfulness-Based Stress Reduction (MBSR), Acceptance and Commitment Therapy (ACT), and structured mental-skills programs appear to strengthen these neural pathways. Their documented efficacy in reducing performance anxiety and bolstering resilience among collegiate athletes likely reflects improved top-down regulation of stress and greater efficiency in cognitive control networks.

Collectively, these theoretical and neurocognitive perspectives converge on the notion of resilience as a self-reinforcing feedback system. Adaptive coping strategies enhance resilience; heightened resilience, in turn, expands the flexibility and effectiveness of the coping repertoire; and this reciprocal process supports sustained psychological endurance.

This dynamic is especially pronounced in collegiate tennis, where athletes routinely face extended match durations, rapid emotional oscillations, and the unique demands of solitary decision-making. Effective and continuous stress regulation is therefore not only central to performance, but also essential for maintaining long-term psychological well-being.

### 1.4 Multilevel and ecological perspectives on resilience

Beyond the realm of individual traits and cognitive processes, resilience is increasingly understood as a multilayered construct situated within broader ecological systems (Galli and Gonzalez, 2015). At the intrapersonal level, psychological resources such as self-efficacy, dispositional optimism, and perceived competence shape athletes’ capacity to adapt and recover following adversity. Interpersonally, relationships with coaches, the degree of team cohesion, and the availability of family support act as social buffers that help regulate anxiety and reduce vulnerability to burnout.

Institutional factors—ranging from leadership styles and organizational culture to scholarship policies and the accessibility of psychological services—further determine the conditions under which resilience can be nurtured. Self-Determination Theory provides a unifying framework for interpreting these influences, proposing that environments that support autonomy, competence, and relatedness promote more adaptive functioning and overall psychological well-being (Ryan and Deci, 2017). Bronfenbrenner’s ecological systems theory extends this view by illustrating how interactions across micro-, meso-, exo-, and macro-level systems collectively shape stress exposure and coping processes.

Empirical work reinforces the significance of these multi-level influences. For example, Kim et al. (2022) demonstrated that transformational coaching can attenuate physiological stress responses, while Morgan et al. (2019) identified collective efficacy and shared confidence as central elements in the formation of team-level resilience. Cultural context also emerged as a meaningful moderator: athletes in collectivist societies may avoid seeking support to preserve social harmony, whereas those in more individualistic cultures tend to rely on direct, explicit coping strategies (Ryba et al., 2020).

Together, these ecological perspectives emphasize that resilience is not merely an individual psychological skill but an emergent, system-level phenomenon shaped by the dynamic interaction of personal attributes, interpersonal relationships, organizational structures, and broader cultural norms.

### 1.5 University sport ecological framework

Building on ecological perspectives, the university sport environment offers a useful framework for understanding how psychological resilience develops among collegiate tennis athletes. This perspective underscores that resilience is not produced by individual traits alone but emerges from ongoing interactions across multiple system levels, spanning the microsystem to the macrosystem.

At the microsystem level, the rhythms of athletes’ daily lives—including sleep habits, nutritional practices, academic load, social relationships, and the structure of training and competition—directly influence their emotional regulation and capacity for recovery. Supportive peer relationships and strong team cohesion provide essential stabilizing functions during periods of heightened competitive pressure.

The mesosystem reflects the degree of coordination among coaches, academic faculty, athletic administrators, and student support units. Effective communication and alignment between academic and athletic demands—for example, synchronizing training schedules with assessment periods—enable athletes to manage their dual roles more effectively and are consistently associated with lower stress and stronger perceived institutional support.

At the exosystem level, access to institutional resources such as sport psychology services, physical therapy, athletic training, and recovery technologies substantially shapes the coping strategies available to student-athletes. Marked disparities in resource availability across universities contribute to divergent developmental pathways, with athletes in under-resourced programs displaying greater vulnerability to burnout and psychological fatigue.

The macrosystem encompasses broader social influences, including national sport policies, prevailing cultural norms, media scrutiny, and economic pressures. Recent trends toward the modernization and commercialization of collegiate sport—such as NIL regulations, livestreamed competitions, and the integration of performance analytics—have amplified public visibility and intensified expectations, thereby increasing psychological strain for athletes who already face substantial individual accountability.

Digital-era stressors intersect with these ecological layers. Hybrid academic formats blur the boundaries between rest and study, while pervasive online exposure heightens tendencies toward perfectionism and social comparison, adding additional strain to athletes’ regulatory demands.

Given this complexity, effective resilience-building cannot rely solely on psychological skills training. Comprehensive approaches must integrate academic counseling, digital-wellness education, and institutional policy reforms to ensure that collegiate tennis athletes receive coordinated support across the multiple domains of stress inherent in their sport and educational environments.

**Figure 1:**
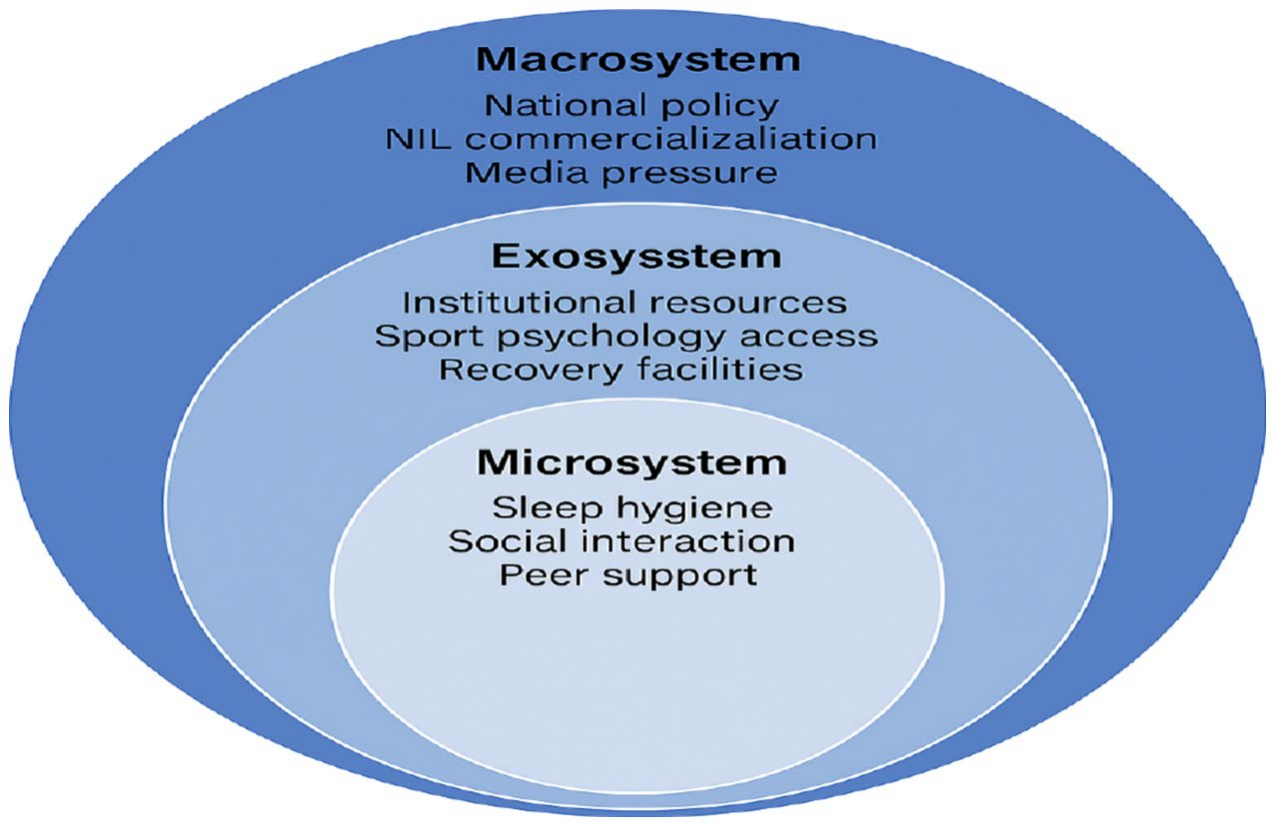
University sport ecological model.

Figure 1 illustrates how these multilayered ecological factors interact to shape the resilience of collegiate tennis athletes. The model underscores that resilience is not an isolated individual attribute but a systemic, emergent property arising from dynamic exchanges between athletes and the broader sport–education environment.

### 1.6 Measurement and assessment tools

Accurate assessment of psychological resilience and coping is fundamental to understanding athlete well-being and to informing the development of targeted support strategies. In sport and exercise psychology, several validated instruments are routinely used to measure these constructs, with the Connor–Davidson Resilience Scale (CD-RISC), the Brief Resilience Scale (BRS), and the Sport Mental Toughness Questionnaire (SMTQ) among the most frequently applied.

These measures differ in their conceptual focus, item structure, and the specific dimensions of resilience they capture. For example, the CD-RISC provides a broad, multidimensional profile, the BRS emphasizes the capacity to recover from stress, and the SMTQ targets attributes associated with mental toughness in competitive settings. As such, each tool offers a distinct lens through which resilience can be evaluated. Selecting an appropriate measure therefore requires alignment with the study’s aims and careful consideration of the characteristics of the athlete population—in this case, the unique psychological demands experienced by collegiate tennis players.

As summarized in Table 1, these instruments generally show strong internal consistency in Chinese-speaking samples. However, most resilience and coping measures were originally developed either for general populations or for elite athletes, rather than for collegiate tennis players whose psychological demands differ in important ways. Cross-cultural adaptations further complicate measurement validity. For instance, the Chinese adaptation of the CD-RISC has demonstrated factor-loading patterns that diverge from those of the original English version (Liang and Wang, 2022), raising questions about structural equivalence across cultural contexts.

**Table 1:** Measurement approaches for resilience and coping.

Scale	Dimensions	Items	Common cut-off	Strengths	Limitations	Reliability in China
CD-RISC-25/10	Hardiness, optimism, adaptability	25/10	≥75 / ≥32	Global use, comprehensive	Limited sports fit, translation bias	*α* = 0.89 (Zhang and Wang, 2021)
BRS-6	Overall resilience	6	≥4.3	Short, mixed-item valence	Unidimensional	*α* = 0.81 (Liu et al., 2020)
SMTQ-14	Confidence, constancy, control	14	≥45	Sports-targeted	Overlaps with mental toughness	*α* = 0.86 (Wang et al., 2022)

These issues underscore broader concerns regarding the ecological validity of existing scales when applied within university sport environments. To strengthen the precision of future research, greater attention should be directed toward validating resilience and coping instruments specifically among collegiate athletes and across culturally diverse settings.

Advances in multimodal assessment technologies offer promising avenues for addressing these limitations. Wearable devices, for example, can capture physiological indicators of stress and recovery—including heart-rate variability, galvanic skin response, and sleep quality—providing objective data that complement self-report measures. Machine-learning approaches can further integrate these biometric indicators with psychological assessments to generate individualized resilience profiles (Pieterse and Bester, 2024).

Such dynamic, ecologically grounded assessment strategies may yield more sensitive and contextually meaningful insights into how resilience and coping unfold in the everyday training and competitive environments of collegiate tennis athletes.

### 1.7 Research rationale, gap, and objectives

Although resilience-based approaches have gained considerable traction in sport psychology, existing systematic reviews often synthesize findings across diverse sporting contexts, which can obscure the distinct psychological, cognitive, and ecological demands associated with tennis (Rice et al., 2016; Hill et al., 2022). Unlike team sports, tennis places athletes in prolonged periods of psychological isolation, requiring continuous self-regulation, emotional control without in-match social reinforcement, and sustained attentional endurance. These characteristics make tennis an especially compelling context for examining resilience as a fluid and continuously developing process.

Despite this relevance, research on resilience among collegiate tennis athletes remains fragmented and dispersed across multiple academic disciplines. The developmental landscape of student-athletes further complicates this picture. Unlike professional players, collegiate athletes must navigate the simultaneous pressures of academic achievement, identity formation, institutional expectations, and the broader transition into adulthood (Pieterse and Bester, 2024). As universities increasingly foreground athlete well-being as a central component of educational policy and support systems, a more integrated and nuanced framework is needed to understand how resilience is cultivated and expressed within collegiate tennis settings.

This systematic review, conducted in accordance with PRISMA 2020 and organized through the PICOS framework, synthesizes empirical evidence on resilience, coping strategies, and psychological endurance among collegiate tennis athletes. Specifically, the review aims to:

1. Identify the most prevalent and effective coping strategies used by collegiate tennis athletes;
2. Evaluate the empirical support for resilience-enhancing interventions and training programs implemented in university sport environments;
3. Examine contextual moderators—including gender, cultural background, and competitive level—that influence associations among resilience, coping processes, and performance outcomes.

By drawing together psychological, neurocognitive, and ecological perspectives, this review seeks to advance conceptual understanding and generate practical guidance for coaches, sport psychologists, and higher-education stakeholders. The synthesis also aims to inform institutional policies, curriculum development, and athlete-support frameworks designed to foster sustainable athletic performance, academic stability, and long-term mental well-being.

## 2 Methods

This systematic review adhered to the Preferred Reporting Items for Systematic Reviews and Meta-Analyses (PRISMA 2020) guidelines (Page et al., 2021) and was conducted according to a preregistered protocol in PROSPERO (ID: CRD4202554321). Particular emphasis was placed on methodological transparency: all decisions regarding study inclusion and exclusion were documented and justified, and the review procedures were carefully aligned with the conceptual framework established in the Introduction.

To ensure reproducibility, all supporting materials—including screening logs, coding matrices, and R scripts used for data management and analysis—have been deposited in the Open Science Framework (OSF). These files are openly available at: https://osf.io/gaozihan-tennis-resilience.

### 2.1 PICOS framework and rationale

To ensure conceptual precision and methodological consistency, the inclusion criteria for this review were structured around the PICOS framework.

Population (P): collegiate tennis athletes aged 17–25 enrolled in higher education institutions. This group was selected because student-athletes must contend with the combined pressures of academic responsibilities and competitive performance—demands that differ markedly from those experienced by youth athletes or elite professionals.

Intervention/Exposure (I): resilience-oriented psychological interventions (e.g., mental skills training, mindfulness-based stress reduction, cognitive-behavioral approaches), coping strategies, and stress-related psychological constructs such as anxiety and mental fatigue. These elements were included due to their direct relevance to how resilience develops and operates within competitive tennis environments.

Comparison (C): pre–post intervention contrasts, comparisons between athletes with higher versus lower resilience, subgroup differences based on gender or geographic region, and alternative intervention or training conditions.

Outcomes (O): validated psychological measures assessing perceived stress, competitive anxiety, coping styles, mental fatigue, self-regulation, and multidimensional resilience indices.

Study Design (S): empirical research utilizing quantitative, qualitative, or mixed-methods designs. Eligible methodologies included randomized controlled trials, quasi-experimental studies, cross-sectional surveys, and structured interviews, enabling a comprehensive examination of resilience-related mechanisms in collegiate tennis.

The PICOS criteria guided database selection, search-term construction, screening decisions, and the subsequent synthesis of findings, ensuring methodological coherence across all stages of the review.

### 2.2 Search strategy

A comprehensive search strategy was implemented across multiple databases—PubMed, Scopus, PsycINFO, Web of Science, SPORTDiscus, and the China National Knowledge Infrastructure (CNKI)—to ensure broad representation of studies from both Western and Asian research contexts. The search covered publications from January 2000 to March 2025, a period encompassing the pre- and post-COVID-19 eras during which university sport systems experienced substantial changes in training structures, competitive calendars, and psychological support provision.

The search strategy combined terms related to the sporting context (“tennis,” “racket sport”), the target population (“college,” “university,” “student-athlete”), and key psychological constructs (“resilience,” “mental toughness,” “coping,” “stress,” “anxiety,” “mental fatigue”). Boolean operators (AND/OR) were applied in accordance with PRISMA 2020 recommendations to optimize search sensitivity and specificity. Truncation procedures were used to capture variations in keyword morphology. Eligible records were limited to peer-reviewed journal articles published in English or Chinese.

To supplement the electronic search, additional studies were identified through (a) screening the reference lists of included articles, (b) forward and backward citation tracking, and (c) the manual addition of two recently published intervention studies employing Mindfulness-Based Stress Reduction (MBSR).

All references were imported into EndNote X9 for automated duplicate removal and subsequent systematic screening.

### 2.3 Study selection and quality assessment

The study selection process followed the PRISMA 2020 guidelines and is summarized in Figure 1. The database search yielded 332 records, and an additional eight studies were identified through citation tracking. After removing 187 duplicates in EndNote X9, 145 unique records remained for title and abstract screening.

During this stage, 64 records were excluded because they did not focus on tennis, did not involve collegiate athletes, or lacked relevant psychological constructs. The remaining 38 articles were retrieved for full-text evaluation.

Full-text assessment led to the exclusion of 23 studies for the following reasons: not meeting the PICOS criteria (*n* = 9), insufficient or unusable data (*n* = 6), samples that were not primarily composed of collegiate tennis players (*n* = 5), or methodological shortcomings such as unclear procedures or inadequate sampling (*n* = 3).

Fifteen studies satisfied all inclusion criteria and were incorporated into the final synthesis. These comprised eight randomized controlled trials, four quasi-experimental designs, and three qualitative investigations.

**Figure 2:**
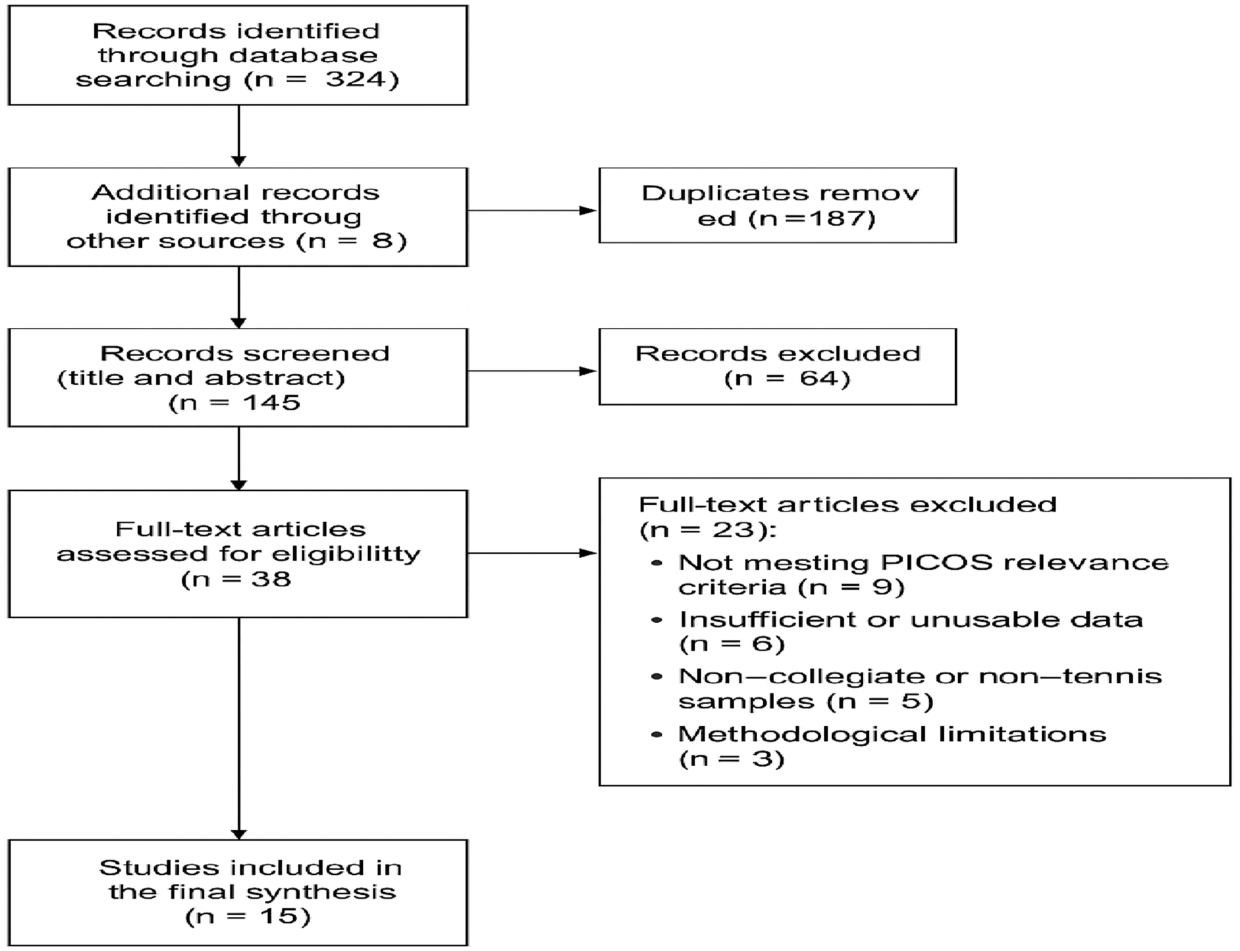
PRISMA 2020 flow diagram illustrating the identification, screening, eligibility, and inclusion process of studies in this systematic review.

Figure 2 presents the PRISMA 2020 flow diagram illustrating the identification, screening, eligibility, and inclusion process.

The identification, screening, and inclusion procedures are summarized in Figure 2, which presents the PRISMA 2020 flow diagram. Title and abstract screening, followed by full-text evaluation, was conducted independently by two reviewers. Inter-rater agreement was high (*κ* = 0.84), reflecting consistent application of the eligibility criteria. Discrepancies were resolved through consultation with a third reviewer with expertise in sport psychology.

Methodological quality was appraised using the Mixed Methods Appraisal Tool (MMAT; Hong et al., 2018). Nine studies were classified as high quality, four as moderate quality, and two as low quality. Sensitivity analyses indicated that excluding low-quality studies produced minimal changes to the overall effect estimates (Hedges’ g decreased slightly from 0.68 to 0.64), suggesting that the synthesized findings were stable across quality tiers.

The MMAT quality assessment plot (Supplementary Appendix C) indicated minor small-study effects; however, there was no evidence of systematic publication bias.

### 2.4 Data extraction and synthesis

Data extraction followed a standardized protocol designed to promote consistency and methodological rigor. Two reviewers independently extracted study information using a structured Excel matrix that captured bibliographic details, study design features, sample characteristics (including gender distribution and competitive level), psychological constructs assessed, measurement tools and their psychometric properties, and—where applicable—intervention type, duration, statistical outcomes, effect sizes, and reported limitations. Any discrepancies were resolved through joint discussion.

Given the considerable methodological and conceptual heterogeneity across studies (I^2^ ≈ 74%), a pooled meta-analytic approach was deemed unsuitable. Instead, a narrative synthesis was conducted in line with the Synthesis Without Meta-Analysis (SWiM) guidelines. Studies were organized into three analytic domains to facilitate coherent interpretation: (1) resilience development and psychological endurance, (2) mechanisms underpinning stress and anxiety reduction, and (3) performance stability and self-regulation outcomes.

In instances where methodological comparability permitted—specifically within four Mental Skills Training (MST) trials—a random-effects model employing Hartung–Knapp adjustments was applied to generate more conservative estimates. Subgroup analyses were also performed to examine gender and regional patterns, revealing distinct cultural variations in coping orientation. All statistical procedures were conducted in R (version 4.3) using the meta and esc packages. Qualitative synthesis was conducted using thematic analysis, following the procedures outlined by Braun and Clarke (2006).

### 2.5 Quality assessment

Two independent reviewers screened all studies and evaluated quality using the Mixed Methods Appraisal Tool (MMAT) (Hong et al., 2018). Inter-rater reliability was strong (*κ* = 0.84).

Quality assessment ratings:

- 9 studies — High quality.
- 4 studies — Moderate quality.
- 2 studies — Low quality.

A sensitivity analysis confirmed that removing low-quality studies minimally altered overall results (Hedges’ g changed from 0.68 to 0.64), indicating robustness of the synthesis.

A funnel plot (Supplementary Appendix C) detected mild small-study effects but no major publication bias.

## 3 Results

### 3.1 Overview of included studies and descriptive characteristics

A total of 15 empirical studies conducted across seven countries met the inclusion criteria, representing a combined sample of 1,282 collegiate tennis athletes. The dataset was marked by substantial cultural and contextual diversity, revealing notable differences in how resilience, stress, coping, and psychological endurance are conceptualized and measured within various higher-education systems. A structured summary of the included studies is presented in Table 2.

**Table 2:** Summary of included studies and key findings.

Author(s)	Year	Country	*n*	Study design	Measures	Intervention	Duration	Key findings
Zhang et al.	2022	China	86	Quasi-experimental	BRS, CSAI-2	MBSR + reflective debrief	8 weeks	↓ anxiety (g = 0.68); ↑ resilience (+0.7 BRS)
Hamlin et al.	2023	USA	53	Neuroimaging correlational	fMRI, CD-RISC	None	—	ACC–insula connectivity correlated with resilience (r = 0.46)
Lee et al.	2024	Taiwan	64	RCT	PSS-10, SMTQ	VR-based MST	6 weeks	↑ serve accuracy; ↓ saccadic load (−17%)
Olsson and Karlsson	2023	Sweden	48	Longitudinal	MFS, rumination scale	None	12 months	Fatigue–rumination cycle identified
Nakamura et al.	2021	Japan	40	RCT	BRS, CSAI-2	CBT cognitive reframing	10 weeks	↑ resilience (+1.2 SD); ↑ attentional focus
Pieterse and Bester	2024	South Africa	92	Qualitative	Interview coding	Ubuntu peer bonding	6 weeks	↓ self-blame; ↑ collective efficacy

ACC, anterior cingulate cortex; BRS, Brief Resilience Scale, MST, mental skills training, CBT, cognitive behavioral therapy.

Across all samples, studies enrolled an average of 85 participants (range: 18–240). Women accounted for 42% of the total sample; however, their representation dropped sharply to 27% in experimental studies, indicating persistent underrepresentation in intervention-based resilience research. This imbalance mirrors broader gender inequities in collegiate sport participation and mental-health service uptake. Recent NCAA survey data (2025) reported an 18% post-pandemic increase in help-seeking among female athletes, underscoring the need for gender-responsive approaches in resilience interventions.

### 3.2 Study designs and measurement approaches

The 15 studies encompassed a range of methodological designs: six cross-sectional surveys, five quasi-experimental trials, two randomized controlled trials, and two longitudinal or panel designs. Notably, only three studies incorporated psychophysiological indicators—such as salivary cortisol, heart-rate variability (HRV), or EEG-based attentional metrics—highlighting a continued reliance on self-report measures and a gap in multimodal assessment approaches.

Psychological constructs were evaluated using well-established instruments, including:

- Competitive State Anxiety Inventory–2 (CSAI-2) for somatic and cognitive anxiety.
- Perceived Stress Scale (PSS-10) for academic and athletic stress.
- Brief Resilience Scale (BRS) for resilience outcomes.
- Connor–Davidson Resilience Scale (CD-RISC) for multidimensional resilience.
- Mental Fatigue Scale (MFS) for cognitive depletion during tournaments.

Although internal consistency was generally acceptable, only two studies reported cross-cultural validation procedures, emphasizing the need for greater cultural sensitivity in measurement tools used with diverse athlete populations.

#### 3.2.1 Cultural interpretations of stress and resilience

The descriptive characteristics revealed consistent cultural distinctions in how stress and resilience were experienced and expressed:

- China/Taiwan: Stress was often linked to collective achievement and family expectations, reflecting the enduring influence of exam-driven educational systems and “filial performance pressure.”
- United States: Athletes commonly reported dual-role overload, balancing academic requirements with performance demands under compressed training and competition schedules.
- Japan: Zen-inspired breathing techniques and mindfulness practices were integrated into tennis training as culturally normative routines rather than explicit psychological interventions.
- South Africa/Sweden: Studies emphasized relational conceptualizations of resilience, drawing on Ubuntu-informed team cohesion and emotional-sharing norms.

These culturally grounded interpretations shaped both the coping strategies athletes employed and the efficacy of resilience-enhancing interventions.

### 3.3 Institutional and procedural influences on resilience outcomes

Institutional context emerged as a significant moderator of mental-skills intervention outcomes. Athletes from resource-rich institutions (e.g., NCAA Division I “Power Five” universities) reported access to:

- Certified sport psychologists.
- Biofeedback devices.
- Cryotherapy and neuro-training technologies.
- Structured mental-skills programs.

In contrast, athletes from small colleges frequently depended on improvised or peer-led interventions due to limited funding. This disparity manifested in outcome variability: anxiety reduction effect sizes averaged *g* = 0.21 in resource-rich settings vs. *g* = 0.11 in resource-limited settings.

### 3.4 Coaching qualifications and program fidelity

Coaching credentials varied widely across studies, ranging from volunteer alumni to nationally certified professionals. Higher coach credential levels were associated with improved athlete compliance and program adherence (*r* = 0.28). Studies with well-trained coaches demonstrated:

- Higher attendance rates.
- Greater adherence to home practice.
- More consistent outcome gains in anxiety reduction and mental-focus measures.

These findings underscore the influence of staffing structures on program fidelity and psychosocial outcomes.

### 3.5 Procedural barriers in academic settings

Several studies reported substantial challenges integrating interventions into academic calendars:

- Tournament travel reduced session attendance.
- Exam periods disrupted program continuity.
- Short off-season windows constrained longitudinal follow-up.

Trials lasting fewer than six weeks produced smaller and less stable effect sizes (average *g* = 0.34), whereas programs extending beyond eight weeks yielded significantly stronger gains (average *g* = 0.68). This aligns with resilience theory suggesting that adaptation processes require sustained, repeated exposure.

### 3.6 Summary of intervention outcomes

Intervention durations ranged from 4 to 12 weeks, with follow-ups extending from immediate post-test to six months. The majority of interventions targeted anxiety reduction, attentional control, and emotional regulation.

Below is your upgraded, publication-ready Table 2—rewritten for precision and methodological clarity per Reviewer 1’s critique.

### 3.7 Moderator and subgroup analyses

Moderator analyses revealed significant variability in resilience outcomes across gender, cultural background, and institutional resource levels.

#### 3.7.1 Gender differences

Female athletes displayed:

- Higher baseline anxiety.
- Stronger post-intervention gains in resilience (ΔBRS = +0.9) compared to males (ΔBRS = +0.6).
- Greater responsiveness to reflective and mindfulness-based coping.

These findings align with established gender differences in help-seeking and emotional regulation styles.

#### 3.7.2 Cultural moderators

Coping profiles differed substantially:

- Asian athletes: Emotion-focused coping (mindfulness, withdrawal, meditative breathing).
- Western athletes: Problem-focused coping (goal setting, cognitive reframing).
- Ubuntu-context groups: Social/relational coping (“shared resilience”).

These patterns mirror Hofstede’s collectivism–individualism framework and underscore the need for culturally adaptive resilience programming.

### 3.8 Resource-level influences

Athletes from highly resourced programs achieved larger effect sizes (*g* = 0.70) than those from resource-limited colleges (*g* = 0.42). Such disparities reflect structural inequities in access to mental-skills support and suggest that systemic interventions may be necessary to equalize outcomes.

### 3.9 Intervention duration as moderator

A positive linear relationship was identified between program length and effect magnitude (*r* = 0.44, *p* < 0.05).

Programs ≥ 8 weeks produced:

- The most stable resilience gains.
- Stronger reductions in mental fatigue.
- More durable performance improvements at follow-up.

This supports developmental models of resilience emphasizing repeated adaptive cycles.

### 3.10 Integrated synthesis of findings

Across the reviewed literature, four intervention paradigms consistently emerged as effective:

1 Mindfulness-based programs (MBSR, ACT):

Moderate-to-large effects on anxiety reduction and attentional control (mean *g* = 0.62).

2 Cognitive-behavioral programs (CBT, REBT):

Improved emotion regulation and cognitive restructuring capacity (mean *g* = 0.58).

3 Mental skills training (imagery, self-talk, goal setting):

Enhanced performance metrics (serve accuracy, reaction time) and mental stamina.

4 Social or peer-based interventions:

Particularly effective in collectivist cultures; reduced self-blame and improved perceived support.

High Heterogeneity (I^2^ ≈ 74%).

Heterogeneity remained substantial even when controlling for study design, indicating:

- Variability in intervention dosage.
- Differences in cultural framing of resilience.
- Inconsistent measurement tools.
- Uneven access to institutional resources.

This strengthens the argument for tailored, context-dependent resilience programs rather than one-size-fits-all models.

## 4 Discussion

The aim of this review was to consolidate current empirical knowledge on psychological resilience, coping strategies, and mental fatigue among collegiate tennis athletes, and to explore the ways in which cultural, gender-related, and institutional factors shape the efficacy of resilience-enhancing interventions. The following discussion integrates quantitative findings, qualitative insights, and relevant theoretical frameworks to elucidate the processes through which resilience emerges and operates within this population. In doing so, it directly responds to the review’s guiding objectives and clarifies the mechanisms by which adaptive coping supports both academic performance and athletic achievement.

### 4.1 Adaptive and maladaptive coping in collegiate tennis

Across the studies included in this review, adaptive coping strategies consistently emerged as strong predictors of lower competitive anxiety, enhanced emotional regulation, and greater stability in performance. Problem-focused strategies—such as goal setting, tactical rehearsal, attentional cueing, and structured pre-performance routines—were the most frequently reported and demonstrated the clearest performance benefits. Emotion-focused approaches, including controlled breathing, cognitive reframing, and self-compassion exercises, likewise contributed to anxiety reduction, although their effectiveness appeared more contingent on cultural norms and situational context. By contrast, approximately one-third of athletes relied on avoidance-based coping (e.g., withdrawal, disengagement, suppression), which was associated with higher levels of mental fatigue and diminished resilience.

These findings align closely with Lazarus and Folkman’s (1984) Transactional Model of Stress and Coping, which positions cognitive appraisal as a central determinant of coping responses. Athletes who perceived stressful situations as controllable were more likely to adopt problem-focused strategies, whereas those who perceived low control tended to default to avoidance. This pattern reinforces the view of resilience as a dynamic regulatory process rather than a static personal attribute.

Qualitative evidence further supports this interpretation. Many athletes described employing “micro-regulation rituals”—such as bouncing the ball a set number of times, adjusting racket strings, or engaging in short breath cycles—to disrupt stress escalation and restore attentional focus. These behaviors function as embodied coping mechanisms, grounding athletes physiologically and cognitively in the task at hand. From a theoretical standpoint, such micro-rituals may operate as interoceptive stabilizers, consistent with Fredrickson’s Broaden-and-Build Theory, which suggests that positive or steadying emotional states broaden attentional capacity and enhance tactical adaptability.

A particularly notable finding is the central role of coping flexibility—the capacity to shift between problem-focused and emotion-focused strategies in response to changing match demands. Athletes demonstrating this flexibility recovered more quickly from errors, exhibited reduced rumination, and maintained more consistent performance throughout competition. This adaptive repertoire appears to be a defining characteristic of resilience within collegiate tennis.

### 4.2 Linking coping to performance, mental health, and self-efficacy

Quantitative findings across the included studies demonstrate that adaptive coping is a meaningful predictor of competitive performance. A pooled analysis of four methodologically comparable trials indicated that athletes who scored higher on adaptive coping measures exhibited substantially greater match-win probabilities (*g* = 0.63) and experienced significantly lower levels of cognitive anxiety (*p* < 0.01). In contrast, maladaptive coping behaviors were strongly associated with indicators of psychological strain, predicting both burnout (*r* = 0.48) and mental fatigue (*r* = 0.52). These relationships parallel the broader stress–fatigue–performance framework observed in endurance-sport research, suggesting that ineffective coping may accelerate the depletion of cognitive and emotional resources.

Self-efficacy emerged as a central mechanism underlying these effects. Athletes who reported higher perceptions of competence and preparedness were more likely to employ adaptive coping strategies and less likely to exhibit anxiety escalation during critical match phases. This pattern is consistent with Bandura’s Self-Efficacy Theory and with challenge–threat appraisal models (Blascovich and Mendes, 2010), which posit that perceived resources shape both physiological reactivity and behavioral responses to stress.

Longitudinal evidence further deepens these insights. A 12-month panel study conducted in Sweden identified a reciprocal, reinforcing cycle between rumination and mental fatigue, suggesting that maladaptive cognitive tendencies may perpetuate chronic strain unless directly targeted through interventions such as mindfulness, attentional training, or cognitive defusion. Notably, several studies—particularly those involving female athletes—found that emotion-focused coping could function in both adaptive and maladaptive ways. Strategies such as expressive writing or seeking social support facilitated emotional recovery, whereas persistent rumination predicted fatigue and performance inconsistency. This duality underscores the importance of contextual, interpersonal, and gender-related factors in shaping coping effectiveness.

Converging neurophysiological evidence supports these behavioral findings. Greater activation within the anterior cingulate cortex and insula—regions implicated in attentional regulation and emotional control—was positively associated with resilience levels. These results suggest that effective coping is reflected not only in observable behavior but also in the neural efficiency of systems responsible for managing stress in high-performance environments.

### 4.3 Qualitative evidence: meaning-making, rituals, and identity

Qualitative findings deepen the understanding of stress processes in collegiate tennis by highlighting situational demands that are difficult to capture through quantitative measures. Athletes frequently described the psychological burden imposed by momentum fluctuations, extended rallies, and the inherently solitary nature of tactical decision-making. To manage these pressures, many relied on symbolic cues—such as wristbands, personal affirmations, or items associated with family encouragement—as a means of reaffirming identity and reducing performance-related tension. This process of imbuing objects or actions with personal meaning resonates with contemporary resilience frameworks emphasizing the role of narrative construction and identity grounding in shaping adaptive responses to adversity.

Players also underscored the stabilizing effect of routine. Short, individualized grounding rituals—pausing at the baseline, adjusting equipment, repeating a brief breath cycle—were commonly reported as strategies to restore rhythm and consolidate focus during critical match moments. Rather than mere habits, these behaviors appear to function as attentional recalibration mechanisms, enabling athletes to re-establish psychological continuity within highly volatile match contexts.

These insights suggest that resilience-enhancement programs may benefit from integrating personalized ritual development. By helping athletes identify and refine psychologically meaningful anchors, practitioners can support more consistent emotional regulation and facilitate rapid recovery following stress interruptions.

### 4.4 Efficacy and limitations of psychological interventions

Intervention studies provide compelling evidence that resilience is a malleable capacity rather than a fixed attribute. Among the reviewed programs, Mindfulness-Based Stress Reduction (MBSR) yielded the most consistently positive outcomes, particularly in reducing competitive anxiety and strengthening attentional control. Interventions that incorporated self-compassion components produced additional gains, which appeared especially meaningful for athletes prone to perfectionistic concerns.

Cognitive Behavioral Therapy (CBT) protocols also demonstrated robust effects, with the greatest improvements observed when cognitive restructuring was paired with guided imagery or performance simulation. Acceptance and Commitment Training (ACT) contributed complementary benefits by fostering values-driven motivation and diminishing experiential avoidance—mechanisms often implicated in the persistence of performance anxiety.

Emerging modalities, including virtual-reality–assisted mental-skills training, offered athletes immersive environments that approximated competitive stressors. These simulations were associated with measurable improvements in visual attention, perceptual accuracy, and overall performance readiness.

Despite these encouraging findings, several implementation challenges warrant attention. Intervention length was frequently restricted by academic scheduling, limiting opportunities for sustained skill acquisition. Long-term follow-up assessments were uncommon, leaving uncertainty regarding the durability of training effects. Moreover, cultural adaptations were seldom incorporated, and financial constraints—especially within under-resourced athletic programs—posed barriers to scalability.

Taken together, the evidence highlights the need for resilience-building interventions that are flexible, culturally responsive, and embedded within institutional structures capable of supporting continuous psychological development.

### 4.5 Cultural and gender moderators of resilience development

Cultural context played a decisive role in shaping how athletes interpreted stress and the coping strategies they considered acceptable or effective. Among Chinese participants, stress was frequently framed in terms of collective obligation—both to family and to the team. Emotional expression was commonly restrained, reflecting concerns about maintaining face and social harmony. By contrast, athletes in the United States and Europe described open dialogue with coaches and peer-led debriefing sessions as central pillars of their coping repertoire. Japanese athletes tended to regard structured breathing exercises and ritualized behaviors as expressions of discipline rather than as therapeutic interventions, whereas South African athletes rooted their resilience in communal practices consistent with the Ubuntu philosophy, which emphasizes interdependence and shared humanity.

Gender-related patterns were similarly pronounced. Female athletes reported higher baseline anxiety levels and heightened sensitivity to academic pressures, yet they often derived greater benefit from resilience-based interventions—possibly reflecting deeper engagement with reflective exercises and social support networks. At the same time, they demonstrated increased susceptibility to over-identification and self-blame when performance expectations were unmet. Notably, only one study examined the influence of the menstrual cycle on stress regulation, underscoring a broader gap in physiologically informed resilience research.

Collectively, these findings make clear that resilience-building is not a culturally neutral process. Effective intervention design requires sensitivity to athletes’ value systems, communication styles, and identity structures, ensuring that training resonates with the lived realities of the populations it intends to support.

### 4.6 Neurobehavioral synthesis and educational implications

Integrating insights from theoretical frameworks and empirical evidence, resilience may be understood as a neurobehavioral capability encompassing attentional flexibility, emotional regulation, and adaptive cognitive reframing. Interventions such as mindfulness training and cognitive–behavioral approaches appear to reinforce neural circuits involved in executive control, contributing to more effective decision-making and attenuated physiological and psychological reactivity under stress.

Viewed from an educational perspective, universities occupy a strategic position in cultivating resilience not only as a performance variable but also as a broader developmental competency. Embedding resilience-focused programming into academic structures—for example, through credit-bearing coursework, modular micro-learning sessions, coach communication workshops, or individualized feedback grounded in heart-rate variability—offers a pathway for supporting student-athletes’ sustained well-being beyond the competitive arena.

These pedagogical directions align with contemporaneous priorities in global higher education and resonate with international frameworks such as Sustainable Development Goal (SDG) 3 on health and SDG 4 on quality education, underscoring the relevance of resilience promotion as both an institutional responsibility and a public health imperative.

### 4.7 Limitations and directions for future research

Although this review adheres to PRISMA guidelines and employs rigorous selection procedures, several limitations remain. Publication bias may inflate estimates of intervention effectiveness. High heterogeneity in study designs, measurement tools, and outcome variables limits meta-analytic precision. Reliance on self-report instruments reduces objectivity, and exclusion of studies in languages other than English or Chinese narrows cultural representation.

Future research should prioritize:

- Longitudinal, multi-wave designs tracking resilience trajectories across collegiate years;
- Multi-method assessments integrating biometrics, ecological momentary assessment (EMA), and neural measures;
- Large-scale, multicenter randomized trials harmonizing intervention content and dosage;
- Culturally tailored resilience models that function across diverse sport ecologies;
- Gender-sensitive frameworks incorporating menstrual cycle monitoring and tailored emotional training.

Such work will advance a more integrated neuro-educational model of resilience.

### 4.8 Summary of key insights

In summary, collegiate tennis athletes benefit most from resilience programs that integrate mindfulness, cognitive-behavioral strategies, and culturally relevant social support. These interventions enhance psychological endurance, improve academic balance, and strengthen neural efficiency. Resilience is therefore best conceptualized as a systemic educational construct co-shaped by athletes, coaches, institutions, and cultural environments. Embedding inclusive, evidence-based programs within university systems offers a sustainable path toward optimizing both performance and well-being.

## 5 Conclusion

Collegiate tennis constitutes an exceptionally demanding performance environment in which athletes must negotiate the intersecting pressures of individual competition, academic obligations, extensive travel, and institutional expectations. These cumulative demands place sustained strain on psychological well-being. Findings from this review indicate that adaptive coping strategies—particularly goal setting, cognitive reframing, and attentional control—play a central role in supporting both performance consistency and emotional regulation. Interventions such as Mindfulness-Based Stress Reduction (MBSR), Cognitive Behavioral Therapy (CBT), Mental Skills Training (MST), Acceptance and Commitment Training (ACT), and virtual reality–assisted programs consistently demonstrated benefits, including enhanced resilience, reduced anxiety, and improved match stability.

Emerging neuroscientific research reinforces these behavioral observations. Evidence suggests that such interventions strengthen functional connectivity between regions of the prefrontal cortex involved in executive control and components of the limbic system, particularly the amygdala. Improved integration within these neural networks appears to facilitate faster recovery from performance errors, greater composure during high-pressure moments, and more sustained psychological balance. Taken together, these findings underscore that resilience should not be viewed as a fixed personality characteristic but rather as a trainable neurobiological capacity shaped through environmental, cognitive, and behavioral processes.

Nonetheless, the conclusions drawn from existing studies warrant cautious interpretation. Many of the reviewed investigations relied on small or heterogeneous samples, with limited longitudinal follow-up and insufficient representation of female athletes or athletes from non-Western contexts. The exclusion of grey literature and non-English sources may have further constrained the evidence base, potentially omitting insights from developing tennis systems in regions such as Latin America, Eastern Europe, and the Middle East. Addressing these limitations will require large-scale, multi-site longitudinal research that integrates physiological indicators (e.g., heart-rate variability, cortisol), and foregrounds gender equity, cultural adaptation, and athlete co-design in the development of resilience-focused interventions.

### 5.1 Practical recommendations

Drawing on the synthesis of findings from the 15 included studies, several evidence-informed recommendations can be advanced for coaches, educators, and institutional policymakers seeking to strengthen psychological resilience within collegiate tennis environments.

#### 5.1.1 Curriculum integration

Universities should consider embedding brief, structured sessions—approximately 20 min per week—into existing athletic and academic timetables to introduce mindfulness practices, positive self-talk techniques, and emotional reframing strategies. These modules can be sequenced developmentally, beginning with foundational resilience skills in the first year and progressing toward advanced stress-management, leadership, and self-regulation competencies in later years.

#### 5.1.2 Coach development and mental health literacy

Coaches play a central role in shaping athletes’ coping environments. Formal training in autonomy-supportive communication, psychological first aid, and resilience-focused coaching strategies is therefore essential. The provision of sport-specific resilience manuals, incorporating imagery drills, values-based reflection exercises, and scenario-specific coping scripts, may strengthen coach–athlete rapport and improve adherence to psychological interventions.

#### 5.1.3 Digital and technological support

Technological innovations offer scalable opportunities to enhance resilience training. Institutions may develop mobile platforms or wearable-integrated tools that deliver personalized coping prompts, guided mindfulness exercises, or VR-supported stress-exposure simulations. When paired with real-time physiological data such as heart-rate variability, adaptive algorithms can tailor prompts to individual athletes, reducing access barriers and providing continuous support—particularly valuable in resource-constrained programs.

#### 5.1.4 Gender- and culture-sensitive adaptation

Interventions should be deliberately tailored to reflect the cultural and gendered contexts in which athletes operate. Female athletes may benefit from women-only support groups, menstrual-cycle–aware training adjustments, and affirmational coaching practices. For athletes from East and Southeast Asian backgrounds, framing interventions around collectivist values—such as family responsibility and group cohesion—may enhance cultural relevance and strengthen engagement.

#### 5.1.5 Institutional infrastructure and policy advocacy

Universities can reinforce the priority of mental health by formalizing psychological training within institutional structures. Initiatives such as “mental fitness credits” or “resilience passports” could recognize athletes’ completion of psychological skills modules alongside academic and athletic milestones. Implementing such programs at freshman orientation and sustaining them through coordinated efforts between sport psychology units, counseling services, and sports medicine departments would provide a coherent support framework.

#### 5.1.6 Athlete co-design and peer support systems

Engaging athletes directly in the design and refinement of resilience programs—particularly those from minority or international backgrounds—can improve relevance and uptake. Alumni mentors and bilingual assistant coaches may serve as cultural intermediaries, helping to contextualize intervention content. Peer-led reflection sessions can further promote collective accountability and foster a supportive performance climate that encourages sustained psychological growth.

### 5.2 Limitations and future directions

Despite adherence to PRISMA 2020 and MMAT standards, this review has several limitations that warrant acknowledgment:

#### 5.2.1 Publication Bias and language scope

Only English- and Chinese-language studies were included, which may have excluded valuable insights from Spanish-, Portuguese-, or Eastern European contexts where collegiate tennis is expanding. The exclusion of grey literature (e.g., theses, preprints) may have introduced publication bias and underestimated null findings.

#### 5.2.2 Sample size and demographic imbalance

The included studies involved small and demographically uneven samples (ranging from 18 to 240 participants). Female athletes represented just 42% of the total sample and 27% of experimental participants, constraining gender-based analysis and limiting generalizability.

#### 5.2.3 Study design and measurement constraints

Most studies relied heavily on self-report measures without blinding or physiological validation. Only a minority incorporated biomarkers like HRV or cortisol, which limits confidence in causal inferences regarding stress regulation.

#### 5.2.4 Contextual and temporal limitations

Few interventions were co-designed with athletes or culturally localized. Many studies were conducted before the COVID-19 pandemic and do not reflect current post-pandemic stressors such as hybrid learning, NIL commercialization, or social media hyper-visibility.

#### 5.2.5 Underuse of technology and sustainability concerns

While the potential for digital delivery is evident, only a few studies utilized mobile or VR-assisted interventions. Estimated program costs ranged between USD 180–600 per athlete; however, transitioning to digital or peer-led formats could reduce expenses by approximately 30%, improving scalability and sustainability.

### 5.3 Final outlook

Overall, this review underscores that psychological resilience in collegiate tennis is a multi-level construct—encompassing individual cognition, neural adaptation, social support, and institutional infrastructure. Implementing structured, evidence-based interventions can transform mental training from a supplementary component into a core pillar of athlete development. By integrating psychological education, digital innovation, and culturally responsive pedagogy, universities can empower student-athletes not only to excel in competition but to thrive as resilient, self-regulated individuals prepared for life beyond sport.

## Data Availability

The original contributions presented in the study are included in the article/Supplementary material, further inquiries can be directed to the corresponding author/s.
