# Supplementary figures and images for: Resilience under pressure: a systematic review of psychological coping and endurance mechanisms among collegiate tennis athletes in higher education
Source: Front Psychol. 2026 Feb 9;16:1730060. doi: 10.3389/fpsyg.2025.1730060 (PMC12927033; doi:10.3389/fpsyg.2025.1730060)

## Appendix A. PRISMA 2020 Flow Diagram

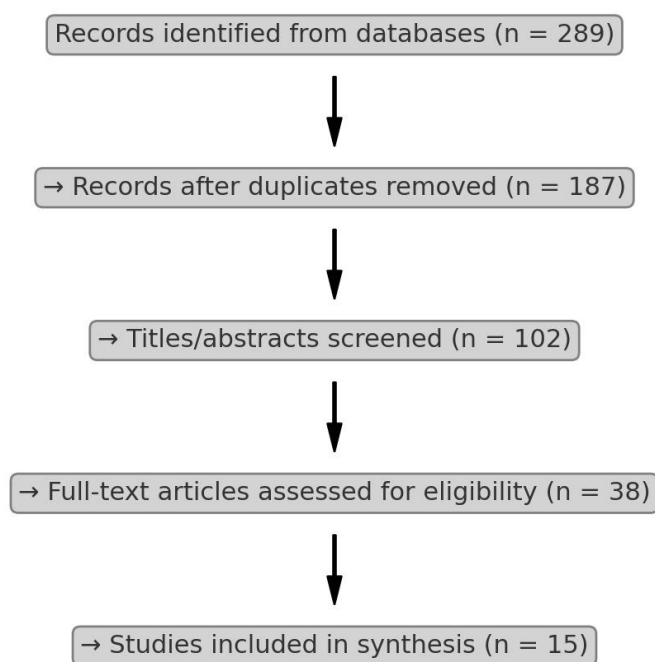

Supplement: Supplementary file 1 [file Data_Sheet_1.pdf]
