# Supplementary material for: Resilience under pressure: a systematic review of psychological coping and endurance mechanisms among collegiate tennis athletes in higher education
Source: Front Psychol. 2026 Feb 9;16:1730060. doi: 10.3389/fpsyg.2025.1730060 (PMC12927033; doi:10.3389/fpsyg.2025.1730060)
Supplement: Supplementary file 2 [file Data_Sheet_2.pdf]

Appendix B. Vote-Counting Table of Included Studies

Study	Country	Intervention	Outcome	Direction	Significance
Zhang et al. (2022)	China	MBSR	↓ Anxiety / ↑ Resilience	Positive	Significant
Hamlin et al. (2023)	USA	None	↑ Neural Activation	Neutral	N/A
Lee et al. (2024)	Taiwan	VR-based MST	↑ Accuracy	Positive	Significant
Pieterse & Bester (2024)	South Africa	Ubuntu Model	↓ Self-blame	Positive	Qualitative
