# Supplementary material for: Resilience under pressure: a systematic review of psychological coping and endurance mechanisms among collegiate tennis athletes in higher education
Source: Front Psychol. 2026 Feb 9;16:1730060. doi: 10.3389/fpsyg.2025.1730060 (PMC12927033; doi:10.3389/fpsyg.2025.1730060)
Supplement: Supplementary file 3 [file Data_Sheet_3.pdf]

Appendix C. MMAT Quality Assessment

Study	Sampling	Data Collection	Design	Analysis	Interpretation	Overall Quality
Zhang et al. (2022)	Yes	Yes	Yes	Yes	Yes	High
Lee et al. (2024)	Yes	Yes	Yes	Partial	Yes	Moderate
Olsson & Karlsson (2023)	Yes	Yes	Yes	Yes	Yes	High
Pieterse & Bester (2024)	Yes	Yes	Qualitative	Yes	Yes	High
